# Adverse Events during Vitreoretinal Surgery under Adequacy of Anesthesia Guidance—Risk Factor Analysis

**DOI:** 10.3390/ph15020237

**Published:** 2022-02-16

**Authors:** Michał Jan Stasiowski, Aleksandra Pluta, Anita Lyssek-Boroń, Seweryn Król, Lech Krawczyk, Ewa Niewiadomska, Jakub Żak, Magdalena Kawka, Dariusz Dobrowolski, Beniamin Oskar Grabarek, Izabela Szumera, Michael Janusz Koss, Anna Missir, Robert Rejdak, Przemysław Jałowiecki

**Affiliations:** 1 Department of Emergency Medicine, Faculty of Medical Sciences in Zabrze, Medical University of Silesia, 41-200 Sosnowiec, Poland; apluta@autograf.pl (A.P.); lech.kraw@gmail.com (L.K.); sernik7@gmail.com (J.Ż.); iza_sz@vp.pl (I.S.); olaf@pro.onet.pl (P.J.); 2 Department of Anaesthesiology and Intensive Therapy, 5th Regional Hospital, 41-200 Sosnowiec, Poland; seweryn.krol@gmail.com (S.K.); aniami521@interia.pl (A.M.); 3 Department of Ophthalmology with Paediatric Unit, 5th Regional Hospital, 41-200 Sosnowiec, Poland; anitaboron3@gmail.com (A.L.-B.); mkawka.buszman@gmail.com (M.K.); 4 Department of Ophthalmology, Faculty of Medicine in Zabrze, University of Technology, Academy of Silesia in Katowice, 41-800 Zabrze, Poland; 5 Department of General, Colorectal and Polytrauma Surgery, Faculty of Health Sciences in Katowice, Medical University of Silesia, 40-555 Katowice, Poland; 6 Department of Epidemiology and Biostatistics, School of Public Health in Bytom, Medical University of Silesia, 41-902 Bytom, Poland; e.j.niewiadomska@gmail.com; 7 Chair and Clinical Department of Ophthalmology, Faculty of Medical Sciences in Zabrze, Medical University of Silesia, 40-760 Katowice, Poland; dardobmd@wp.pl; 8 Department of Histology, Cytophysiology and Embryology, Faculty of Medicine in Zabrze, University of Technology, Academy of Silesia in Katowice, 41-800 Zabrze, Poland; bgrabarek7@gmail.com; 9 Department of Gynaecology and Obstetrics, Faculty of Medicine in Zabrze, University of Technology, Academy of Silesia in Katowice, 41-800 Zabrze, Poland; 10 Augenzentrum Nymphenburger Höfe, 80335 Munich, Germany; michael.koss@me.com; 11 Department of Ophthalmology, Augenklinik der Universität Heidelberg, 69120 Heidelberg, Germany; 12 Department of General Ophthalmology, Medical University of Lublin, 20-059 Lublin, Poland; robert.rejdak@umlub.pl

**Keywords:** pars plana vitrectomy (PPV), general anesthesia (GA), postoperative nausea and vomiting (PONV), surgical pleth index (SPI), oculo-cardiac reflex (OCR), adequacy of anesthesia (AoA)

## Abstract

Vitreoretinal surgeries require the administration of general anesthesia (GA) in selected groups of patients. The administration of intraoperative rescue narcotic analgesia (IRNA) during GA poses the risk of postoperative nausea and vomiting (PONV). The surgical pleth index (SPI), a crucial component of the adequacy of anesthesia (AoA) guidance of GA, optimizes the intraoperative titration of IRNA. The current analysis evaluated the risk factors for the occurrence of PONV and the oculo-cardiac reflex (OCR) in patients undergoing pars plana vitrectomy (PPV) under AoA guidance. In total, 175 patients undergoing PPV were randomly allocated to receive either GA with SPI-guided IRNA administration using fentanyl alone or in addition to different preoperative analgesia techniques. Any incidence of PONV or OCR was recorded. Obesity, overweight, smoking status, motion sickness, postoperative intolerable pain perception, female gender, fluid challenge and arterial hypertension did not correlate with an increased incidence of PONV or OCR under AoA guidance. Diabetes mellitus, regardless of insulin dependence, was found to correlate with the increased incidence of PONV. The AoA regimen including SPI guidance of IRNA presumably created similar conditions for individual subjects, so no risk factors of the occurrence of PONV or OCR were found, except for diabetes mellitus. We recommend using AoA guidance for GA administration to reduce OCR and PONV rates.

## 1. Introduction

Vitreoretinal surgeries (VRS) are increasingly common operations in ophthalmology due to the expansion of the geriatric and diabetic populations. Although in many centers VRS are performed under regional anesthesia (RA) accompanied by monitored anesthesia care (MAC) [1,2], there is a growing number of patients (especially among the elderly) who require immobilization on the operating table under general anesthesia (GA) [3]. GA alone is, however, very often associated with adverse complications due to the use of intraoperative rescue narcotic analgesia (IRNA), which was identified as a risk factor for postoperative nausea and vomiting (PONV) in the first 24 h after VRS. Therefore, the use of IRNA should be avoided when possible, as any incidence of PONV can reduce patients’ comfort and satisfaction, and anesthesia may also lead to increases in intraocular pressure and blood pressure, deteriorating the effect of the performed VRS (wound dehiscence, iris prolapse and intraocular bleeding) [4]. On the other hand, insufficient intraoperative analgesia is supposed to lead to life-threatening hemodynamic disturbances, the occurrence of oculo-cardiac reflex (OCR) and increased postoperative pain perception. Therefore, in order to minimize this negative impact, different techniques of pre-emptive analgesia (PA) are employed, which can minimize the necessity of IRNA administration.

The use of IRNA should be rational but monitoring the efficacy of analgesia remains a challenge during GA, as volatile anesthetics have been proven to blunt the hemodynamic effect of surgical stimulation expressed as increases in heart rate and blood pressure [5]. Bergmann et al. [6] reported fewer adverse events, a reduced demand for IRNA and shorter emergence from anesthesia when the titration of IRNA was based upon the observance of Surgical Pleth Index (SPI) fluctuations, which is a crucial component of the adequacy of anesthesia (AoA) concept of the guidance on general anesthesia (GA) [7]. SPI is derived from the photoplethysmography waveform amplitude and the heartbeat-to-beat interval. Several studies have proven that fluctuations in the SPI value reflect the nociceptive–anti-nociceptive balance [8,9]. Changes in the SPI value are reported to correspond to the serum opioid concentration [10], so SPI guidance seems to be an excellent tool for the administration of intraoperative RNA as the display of its fluctuations on a monitor in digital form (0—no pain; 100—maximum pain) facilitates its intraoperative use and interpretation [5,6].

In our previous studies, we investigated the influence of the AoA guidance on GA in patients undergoing pars plana vitrectomy (PPV) on the rate of incidence of postoperative intolerable pain perception (PIPP), hemodynamic stability [11], and the rate of incidence of OCR and PONV [12]. The current analysis aimed to identify risk factors for the occurrence of OCR or PONV when AoA guidance for GA alone or combined with different PA techniques was utilized.

## 2. Results

The analysis in this study involved a total of 175 patients: 97 (55.4%) women and 78 (44.6%) men. The patients were divided into five equal groups: patients who received general anesthesia (GA); patients who received preoperative analgesia (PA) using metamizole (M); patients who exhibited preprocedural peribulbar block (PBB group) using a mixture of lignocaine and bupivacaine with Hamilton’s technique 1 min before induction of GA; patients who exhibited preprocedural peribulbar block (PBB group) using paracetamol (P); patients who received preventive topical analgesia by triple instillation of 2% proparacaine (T). Each group contained 35 patients (20%). The detailed characteristics of patients’ anthropometric data are shown in the supplementary materials (Table S1). The statistical analysis showed no statistically significant differences in the anthropometric data (*p* > 0.05).

In the first step of our study, we analyzed the rate of postoperative pain perception, OCR and PONV in patients in accordance with their group allocation. It was observed that the highest number of patients with NRS > 3 was in the M and T groups (eight patients; 22.9%), whereas in the GA and P groups, this was six patients (17.1%), and five patients in the PBB group (14.3%). The differences were not statistically significant (*p* > 0.05). The statistical analysis showed significantly higher fentanyl (FNT) values in the M group (165.7 ± 116.8 mg) in comparison to other groups (GA—144.3 ± 102.7 mg; *p*—95.7 ± 81.7 mg; PBB—95.1 ± 101.3 mg; T—148.6 ± 120.3 mg; *p* < 0.05). In addition, the performed analysis indicated that regardless of the PA technique used there were relatively small and statistically insignificant negative postoperative reactions—PONV and OCR. The rate of OCR was assessed in each group (M—six patients; GA group—four patients; P and PBB groups—four patients; T—three patients; *p* > 0.05) as well as PONV (M—six patients; GA, P and T group—four patients; T—three patients; *p* > 0.05).

The mean demand for IRNA had no significant impact on the occurrence of PONV and OCR, despite the group allocation, although a significantly higher demand for intraoperative IRNA using FNT was recorded among patients in the M group as compared to the PBB group (Table 1).

**Figure 1:**
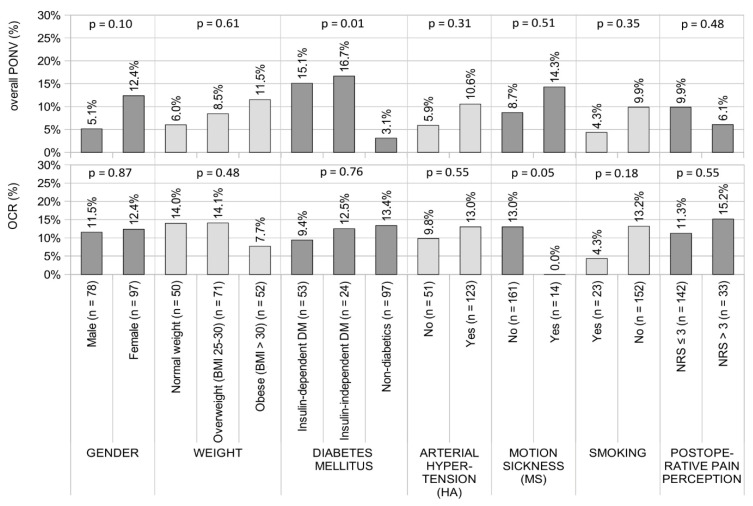
Results of the relationship between gender, BMI, diabetes mellitus, arterial hypertension, motion sickness, smoking status, NRS > 3 and the occurrence of PONV and OCR. Results presented as percentages for nominal variables. *p*-values obtained by the X^2^ test for nominal variables; pairwise comparison of proportions: significant differences in percentages between the non-DM and DM groups (*p* < 0.05). Abbreviations: PONV—postoperative nausea and vomiting; OCR—oculocardiac reflex; NRS—numeric pain rating scale.

In the last stage of our research, we analyzed whether gender, BMI, diabetes mellitus, motion sickness or smoking status had any influence on the occurrence of PONV, OCR and NRS > 3. The data are shown in Table 2 and Figure 1. The performed statistical analysis indicated that only the relationship between diabetes mellitus and PONV risk was statistically significant (*p* < 0.05). We did not observe any significantly different correlations between the analyzed parameters (*p* > 0.05).

## 3. Discussion

Incidences of intraoperative OCR and PONV both constitute a challenge for the anesthesiologist intraoperatively and in the PACU, as well as for the staff of the department of ophthalmology postoperatively. Therefore, there have been attempts made to optimize the anesthetic regimen in order to minimize their occurrence and identify the risk factors for their presence in order to provide the individuals at risk with even more extensive care and supervision [13].

The utility of the adequacy of anesthesia (AoA) technique of intraoperative monitoring (SPI alongside SE and e-NMT) created a comparable anesthetic regimen for each individual patient included in the current study. The utility of monitoring the depth of anesthesia using state entropy (SE) meant that every patient was subjected to almost the same concentration of volatile anesthetic (leading to similar suppression of brain activity).

As a result of the steady suppression of brain activity expressed using SE within the target value of 40–45, each patient was expected to perceive the painful surgical stimulation during PPV with the same intensity; when the intravenous or regional PA was insufficient, ∆SPI > 15. The utility of SPI to guide IRNA administration, contrary to IRNA guidance based on the anesthesiologic intuition and observance of hemodynamic changes, created comparable conditions to administer IRNA to individual patients, as IRNA administration based on changes in the SPI value was proven to correspond to the serum opioid concentration [10]. Therefore, the employment of AoA enabled the analysis of risk factors of the occurrence of PONV and OCR, as under such an anesthetic regimen only individual characteristics could presumably be responsible for their presence and enabled a reliable risk factor analysis. Numerous reliable PA regimens were successfully undertaken to eradicate PONV and ensure a smooth postoperative recovery [1,13,14,15,16].

Based on the current literature, numerous studies were performed to identify the risk factors of the occurrence of PONV. Female gender, smoking status, motion sickness, the predictive use of narcotic analgesics (constituting Apfel score), history of DM (insulin dependent or insulin independent), obesity, and anesthetic regimen were considered to be potential risk factors for the occurrence of PONV [17].

In the study of Nitahara et al. [18] concerning risk factors for the occurrence of PONV after PPV, female gender, lower BMI and inhalational anesthesia were found to constitute the main risk factors for the occurrence of PONV after PPV performed in adults. In their study, female patients were also found to have a 3.1–5.8 times higher risk of the occurrence of PONV in comparison to male patients, and this gender difference was found to constitute the only factor influencing the incidence of PONV throughout the period of observation in their study. They observed the incidence of nausea in 16 males and 41 females and incidences of vomiting in 11 males and 26 females out of 247 patients, meeting the inclusion criteria for this study, as compared to 4 males and 12 females out of 175 patients in the current study, which was not statistically significant.

In the study of Iwamoto et al. [19], female gender was found to be a risk factor of PONV, regardless of the type of ophthalmic surgery, similar to the findings of several other studies [20,21,22], and they found droperidol to be an efficient antiemetic agent in the group of female patients. Similar to the observations of Apfel et al. [23], they also found that inhalational anesthesia correlated with increased incidence of nausea, observed in 24 cases compared to 6 incidences in propofol anesthesia. Propofol was found to reduce the rate of incidence of PONV due to its direct antiemetic properties [24] that are widely used to prevent PONV in patients undergoing thyroidectomy [25], cesarean section [26] and laparoscopic cholecystectomy [27]. Nevertheless, during maintenance of GA we used sevoflurane due to its pre-conditioning properties of ameliorating myocardial ischemia/reperfusion injury in diabetic patients [28] who constituted about 44% of patients in our study.

Despite the group allocation, in the current study, sevoflurane was used to conduct GA, resulting in an overall incidence of PONV in a total of 9% of patients, which is a relatively satisfying result.

There are conflicting data regarding the influence of overweight or obesity on the rate of incidence of PONV. Watcha et al. [29] hypothesized the incidence of PONV to be increased in obese subjects due to possible causative factors, such as a larger volume of adipose tissue, larger residual gastric volume and difficulties in mask ventilation, contrary to the findings of Apfel et al. [23] and Kranke et al. [25], who did not observe any relationship between increased BMI and the rate of incidence of PONV [25]. As only 1.6% of subjects were obese in the study of Nitahara et al. [18], which showed a decreased incidence of PONV in this group of patients, in contrast to 10.1% in the review of Kranke et al. [30], the conclusion regarding no influence of BMI > 30 on the rate of PONV is convincing. Kim et al. [31], in their study of 113,881 patients who underwent GA, showed that an increased BMI reduced the incidence of PONV [26], whereas being underweight did not. In the current study, the number of overweight patients was 72 (41.4%), whereas obesity was noted in 52 (29.9%). Therefore, the relatively high percentage of patients with abnormally increased weight, as compared to the abovementioned literature, might have contributed to the final low incidence of low overall PONV, supporting the theory of Kim et al. [31].

Numerous studies report the incidence of PONV to be decreased in patients with smoking status [20,32,33,34,35]. In the current study, only one smoker and 13 non-smokers developed PONV, as compared to 22 and 139 who did not, respectively, out of 175 subjects, which was not statistically insignificant. Our study findings are in concordance with the conclusion of Nitahara et al. [18] that smoking status has no predictive value for the occurrence of PONV when undergoing PPV. In their study, 42 non-smokers had nausea and 27 experienced vomiting out of 247 subjects, which was not statistically significant [18].

There are conflicting data concerning the influence of the administration of IRNA on the rate of incidence of PONV and OCR. Some reports identify IRNA as increasing the risk of the presence of PONV [36,37], as after IRNA administration approximately 40% of patients were found by some authors to be likely to experience nausea whereas 15–25% of patients experienced vomiting [14,38,39]. In other reports, meanwhile. such a correlation was not found, irrespective of the IRNA in question [40].

In ophthalmic surgery, the use of IRNA was identified as a risk factor for the presence of PONV by Mandelcorn et al. [41], who observed that postoperative nausea occurred almost three times more frequently among patients undergoing PPV under RA with conscious sedation who received IRNA as compared to patients who presented no such requirement. They concluded that the utility of IRNA was the only factor responsible for nausea in the postoperative period [41]. In the current study, such a correlation was not found. In our study, no correlation was found between the demand for IRNA and the incidence of PONV and OCR. To our surprise, the demand for IRNA was the highest among patients receiving metamizole, but the rate of incidence of PONV was the lowest in this group, similarly to patients receiving paracetamol, whose demand for IRNA was much lower, but a statistically significant difference was not found.

The declaration of PIPP, as understood by NRS > 3, also did not correlate with the rate of incidence of PONV in the current analysis. Contrary to our study findings, the reduction in the rate of incidence of PONV correlated significantly with the reduction in the pain score and not with reduced opioid use related to paracetamol application in the study of Apfel et al. [41]. They suggested either a direct link between the analgesic effect and PONV reduction or a direct effect of paracetamol on PONV, similarly to the observation made by Cok et al. [42] in children undergoing strabismus surgery who recorded a decreased rate of incidence of PONV during the first 24 h of receiving intraoperative intravenous paracetamol. Additionally, motion sickness did not correlate with the rate of incidence of PONV in the current analysis.

We also analyzed the possibility of coincidence between the two most common comorbidities of patients undergoing vitreoretinal surgeries, arterial hypertension and DM, in relation to the incidence of PONV [43,44,45]. Diabetic patients benefit from enhanced vision and improved lifestyle from the introduction of vitreoretinal surgeries [46].

In the current study, the history of DM, regardless of dependence on insulin, was identified as an independent risk factor for the incidence of PONV. Symptoms of PONV are classic and frequent gastroparesis following DM [47]. We suppose that this due to the fact that diabetic patients mainly suffer from nausea in the morning before eating, during the night and when not eating [48], which resembles a perioperative fasting regimen. PONV in these patients might have occurred either as a coincidence or as an immediate postoperative adverse event, induced by the delayed effect of IRNA in individual cases with diabetic autonomic neural imbalance progressing to diabetic autonomic neuropathy, delaying gastric emptying [49]. No correlation between the presence of HA and incidence of PONV was found, regardless of the group allocation.

According to the current literature, there are conflicting data concerning the influence of fluid management in the perioperative period on the rate of presence of PONV [50,51,52]. The perioperative administration of an adequate volume of intravenous fluid was reported to be able to correct the intravascular volume deficits, finally reducing PONV [53,54].

Apfel et al. [53] concluded in their review that the supplemental intravenous administration of crystalloids played a key preventive role in the management of PONV, which is an inexpensive and non-pharmacological modality that reduces the incidence rate of PONV that is free from the observable side effects of pharmacological therapy [53]. Mallick-Searle et al. [37], in their study concerning children undergoing strabismus surgery, observed the superiority of combined dexamethasone and super-hydration over each therapy alone in terms of reducing the rate of incidence of PONV [37].

Although the mechanism underlying the reduction in PONV achieved by supplemental fluid therapy remains unclear, Ismail et al. [55], in their study concerning the prophylaxis of PONV, hypothesized that during elective surgery overnight fasting in addition to intraoperative fluid loss results in hypovolemia and a subsequent reduction in blood flow to the gut. In their opinion, the correction of gut ischemia suppresses the excessive serotonin release, which can possibly become a trigger of PONV. Therefore, the improvement of mesenteric perfusion by the prevention of gut ischemia and a decrease in serotonin release may possibly add to the reduction in the rate of presence of PONV [55].

In the current study, there were no statistically significant differences in perioperative fluid challenge between studied groups and no correlation between fluid challenge and the abovementioned risk factors was found. First, all patients received a fixed volume of crystalloids (10 mL/kg of body weight) to balance the overnight fluid loss, which was estimated to be approximately 40 mL/hour. Intraoperatively, when the depth of anesthesia using spectral entropy EEG (SE) was fixed at [40,41,42,43,44,45] in all patients and the IRNA was kept under SPI guidance levels (in accordance with AoA regimen), the use of anesthetic agents had a presumably similar impact on the intraoperative fluid requirement in individual patients, despite the group allocation. We hypothesize that the intraoperative monitoring of the depth of anesthesia using SE and analgesia using SPI could have created comparable conditions for all patients to direct fluid challenge according to their needs based on the intraoperative hemodynamic changes in response to the anesthetic regimen required to perform PPV.

The OCR is defined as an HR decrease of 20% from the baseline value or if dysrhythmias or sinoatrial arrest occurs during ocular manipulation [29] requiring atropine administration if the HR fails to increase to a baseline value after the release of surgical stimulation on the demand of the anesthesiologist. In the current study, the total rate of incidence of OCR was 12% (21 subjects out of 175), regardless of group allocation. Contrary to the observations of Ruta et al. [56] and Sajedi et al. [57], who observed a reduced rate of incidence of OCR when topical anesthesia was added to GA in patients undergoing PPV, in our study AoA guidance of GA completely blunted the effect of pre-emptive analgesia techniques. Moreover, no correlation between the rate of incidence of OCR and the abovementioned risk factors of its incidence was found. We presume that the employment of an anesthetic modality based on AoA guidance was responsible for the elimination of any cofounding factors of its incidence, similar to the case of PONV.

There are some limitations to the current analysis. First, nausea is a subjective phenomenon, which may be underreported by patients, especially in the population of diabetic patients, who may either misinterpret nausea as general illness after GA or may be so habituated to everyday nausea that they do not report it postoperatively. Second, despite the group allocation, patients were premedicated using midazolam as a part of the anesthetic regimen, which was proven to decrease the rate of PONV after PPV [58]. Its administration could have proportionally affected the final rate of incidence of PONV. Third, as shown in the current literature, there is no consistent algorithm for the titration of IRNA available, which could be due to the on-line observation of fluctuations in the SPI value [59]; therefore, we adopted a methodology of IRNA titration from our previous studies [60,61,62,63,64].

## 4. Materials and Methods

The study was conducted according to the guidelines of the Declaration of Helsinki and approved by the Institutional Review Board of the Bioethical Committee of the Medical University of Silesia (protocol code KNW/0022/KB1/101/15 and date of approval 29 September 2015). The project was registered in the Clinical Trial Registry (SilesianMUKOAiIT2, NCT02973581) and received the same approval as previous studies [11,12,60,61]. Informed consent was obtained from all patients recruited. This section builds upon our previous work [11,12,60,61].

### 4.1. Subjects

A total of 200 patients with indications for vitrectomy were invited to take part in the study. Patient data were stored in accordance with applicable laws in the Department of Anesthesiology and Intensive Therapy of the 5th Regional Hospital in Sosnowiec, Poland. Randomization was conducted via opening sealed envelopes after acquiring written informed consent. Before starting the analysis, the patients’ data were deleted so that individual patients could not be identified. The group size was estimated on the basis of the total number of vitrectomy procedures performed at the 5th Regional Hospital in Sosnowiec, Poland, which is 333 per year, and the confidence level of 95% and margin of error of 5%. Finally, 175 patients who underwent vitrectomy were enrolled in this study. They were divided into five groups according to the anesthesia used during the procedure. According to our previous articles, the groups were characterized as (1) the GQ group, including patients who received general anesthesia alone; (2) the T group, including patients who received preventive topical analgesia by the triple instillation of 2% proparacaine (alcaine, propacaine hydrochloride ophthalmic solution USP 0.5%, 15 mL, Sandoz a Novartis Company, Warszawa, Poland) 15 min before the induction of GA; (3) the PBB group, including patients who received PBB using a mixture of 3.5 mL each of 2% lignocaine (Lignocainum hydrochloricum WZF 2% solution, 20 mg/mL, 2 mL, Polfa Warszawa S.A, Warszawa, Poland) and 0.5% bupivacaine (Bupivacainum hydrochloricum WZF 0.5%, 5 mg/mL, 10 mL, Polfa Warszawa S.A) with Hamilton’s technique 1 min before the induction of GA [40]; (4) the M group, including patients who received PA using a single dose of 1 g of metamizole (Pyralgin 0.5 g/mL, 5 mL solution; Polpharma SA, Starogard Gdański, Poland) in 100 mL of saline solution intravenously 30 min before arrival at the operating room; and (5) the P group, including patients who received PA using a single dose of 1 g of acetaminophen (Paracetamol Kabi 10 mg/mL, solution 100 mL; Fresenius Kabi, Kutno, Poland) in 100 mL of saline solution intravenously 30 min before arrival at the operating room. The following vital parameters were monitored both during induction of anesthesia and throughout the surgical procedure: (1) non-invasive arterial pressure (NIBP), (2) HR, (3) standard electrocardiography, (4) pulse oximetry (SaO2), (5) fraction of inspired oxygen in the gas mixture, (6) fraction of inspired sevoflurane (FiAA), (7) fraction of expired sevoflurane, (8) EtCO2, and (9) minimal alveolar concentration of sevoflurane. [11,12,60,61].

### 4.2. Assessment of the Surgical Pleth Index

EEG entropy (state and response) was used to monitor the depth of anesthesia, while the SPI index was used to assess intraoperative analgesia. In addition, the neuromuscular blockade (Carescape B650, GE, Helsinki, Finland) was analyzed. The average SPI value and the calibration of the SPI sensor was possible thanks to the monitoring of the SPI value, which started 5 min after putting on the laryngeal mask and continued until the orbital sterilization began. At the intraoperative stage, the SPI value was read at 1 min intervals. In a situation where the SPI value increased by more than 15 compared to the value in the preoperative stage (∆SPI > 15), the patient was administered 1 µg/kg of FNT intravenously every 5 min, until the SPI value dropped to the mean value from the preoperative stage. We took a ∆SPI of >15 as the threshold at which emergency analgesia should be applied. The adoption of a difference value ∆SPI > 15 as an indication for emergency analgesia allowed us to minimize the risk of administering too much FNT, which may result from fluctuations in the SPI value and possible incorrect SPI calculations [11,12,60,61].

### 4.3. Assessment of the Occurrence of OCR and PONV

OCR incidence was monitored during vitrectomy, while PONV incidence was monitored both during surgery and for the first 24 h after surgery. “In the event of bradycardia, 0.5 mg of atropine (Atropinum sulfuricum WZF 1 mg/mL, solution 1 mL, Polfa Warszawa SA) was used, and in the case of hypotension, 5 mL of crystalloid per kg of body weight and a single dose of 5 mg of ephedrine (Ephedrinum hydrochloricum WZF 25 mg/mL, 1 mL of solution, Polfa Warszawa SA) [11,65]”. The indicators of SPI, HR, systolic arterial pressure (SAP), mean arterial pressure (MAP), diastolic blood pressure (DAP) and SaO_2_ were used to monitor the health of patients in the postoperative period, in accordance with the recommendations of Abouammoh et al. [66]. A numeric pain rating scale (NRS) was used for each patient’s pain rating. When the NRS score was below 3, standard non-steroidal anti-inflammatory drugs were administered in accordance with the Polish Society of Anaesthesiologists [67]. As mentioned in the previous work, “both NRS and SPI values were noted for severe (NRS 7–10), moderate (NRS 4–6), or mild pain (NRS 0–3) perception intervals [11]”. The risk of PONV was also assessed using the Apfel scale, which considers the following risk factors: female gender, history of motion sickness or PONV, no smoking, and postoperative opioid use. The Apfel score was determined before the anesthetic procedure. Depending on the absence of 1–4 of these risk factors, the incidence of PONV is 10%, 21%, 39%, 61% and 79%, respectively [11,45,59].

### 4.4. Statistical Analysis

Statistical analysis was performed using the STATISTICA 13PL software (StatSoft Sp. z o.o., Cracow, Poland) with a statistical significance threshold of *p* < 0.05. We performed one-way analysis of variance (ANOVA) and if the test showed statistically significant differences between variables, Tukey’s post hoc test was used. On the other hand, when the assumptions of the Shapiro–Wilk test were not met we used the non-parametric Kruskal–Wallis test followed by Dunn’s post hoc test. The Chi-square (X^2^) Test was used to analyze the independence of data expressed on a nominal scale. Moreover, to assess the relationship between the occurrence of OCR, PONV and analyzed risk factors, the crude OR (odds ratio), and 95%CI (confidence interval) with Fisher’s exact test were used.

## 5. Conclusions

The analysis in the current study revealed that DM, regardless of insulin dependence, constituted the only risk factor of the incidence of PONV. Female gender, smoking status, motion sickness, overweight and obesity, declaration of PIPP (NRS > 3), dose of IRNA and arterial hypertension were not found to correlate with the presence of intraoperative OCR and/or PONV. We assume that the utility of AoA monitoring created comparable conditions in the anesthetic regimen for each patient in the study, which blunted the influence of already-identified risk factors for the incidence of PONV in the current literature.

## Figures and Tables

**Table 1:** Comparison of intraoperative FNT requirement in patients with incidence of PONV and OCR according to group allocation.

Parameter	Total *n* = 175 (100%)	GA Group *n* = 35 (20%)	M Group *n* = 35 (20%)	P Group *n* = 35 (20%)	PBB Group *n* = 35 (20%)	T Group *n* = 35 (20%)	*p*-Value
Total *n* = 175 (100%)	129.9 + 108.2 100 (150)	144.3 + 102.7 150 (150)	165.7 + 116.8 200 (200)	95.7 + 81.7 100 (50)	95.1 + 101.3 50 (150)	148.6 + 120.3 150 (200)	*p* = 0.02 M vs. PBB *
Mean intraoperative FNT requirement in patients with overall PONV	143.8 + 99.8 175 (200)	137.5 + 62.9 150 (75)	250 + 0 250 (0)	150 + 0 150 (0)	100 + 141.4 100 (200)	143.8 + 120.8 200 (250)	*p* = 0.65 NS
Mean intraoperative FNT requirement in patients without overall PONV	128.5 + 109.3 100 (150)	145.2 + 107.5 150 (150)	163.2 + 117.6 175 (200)	94.1 + 82.4 100 (50)	94.8 + 101.4 50 (150)	150 + 122.5 100 (200)	*p* = 0.08 NS
*p*-value	*p* = 0.48 NS	*p* = 0.94 NS	-	-	-	*p* = 0.83 NS	*p* = 0.99 NS
Mean intraoperative FNT requirement in patients with OCR	127.6 + 96.5 100 (150)	100 + 70.7 75 (100)	141.7 + 91.7 150 (100)	87.5 + 47.9 75 (75)	82.5 + 88.8 65 (135)	250 + 132.3 200 (250)	*p* = 0.25 NS
Mean intraoperative FNT requirement in patients without OCR	130.2 + 110 100 (150)	150 + 105.7 150 (150)	170.7 + 122.1 200 (200)	96.8 + 85.6 100 (50)	96.8 + 104 50 (150)	139.1 + 116.9 100 (200)	*p* = 0.04 M vs. PBB *
*p*-value	*p* = 0.91 NS	*p* = 0.42 NS	*p* = 0.69 NS	*p* = 0.96 NS	*p* = 0.89 NS	*p* = 0.21 NS	*p* = 0.34 NS

Results presented as mean ± standard deviation; NS—statistically insignificant differences (*p* > 0.05); *—statistically significant differences (*p* < 0.05 by the Kruskal–Wallis test (non-parametric one-way ANOVA test) for quantitative variables or the post hoc tests); GA group—patients who received general anesthesia; M group—patients who received PA using a single dose of 1 g of metamizole intravenously 30 min before arrival at operating room; P group—patients who received PA using a single dose of 1 g of acetaminophen intravenously 30 min before arrival at the operating room; PBB group—including patients who received PBB using a mixture of 3.5 mL each of 2% lignocaine and 0.5% bupivacaine with Hamilton’s technique 1 min before induction of GA; T group—patients who received preventive topical analgesia by triple instillation of 2% proparacaine; PONV—postoperative nausea and vomiting; OCR—oculocardiac reflex; SD—standard deviation; IQR—interquartile range [11,12].

**Table 2:** Crude odds ratio and its 95% confidence interval (CI) for occurrence of OCR and PONV according to analyzed risk factors, despite group allocation.

Parameter	Gender		Weight			
	Male	Female	Normal Weight	Overweight (BMI 25–30)		Obese (BMI > 30)
non-PONV	74 (94.9)	85 (87.6)	47 (94)	65 (91.5)		46 (88.5)
overall PONV	4 (5.1)	12 (12.4)	3 (6)	6 (8.5)		6 (11.5)
OR (95%CI) *p*-Value	ref	OR = 2.61 (0.81–8.44) *p* = 0.1 NS	ref	OR = 1.45 (0.34–6.08) *p* = 0.61 NS		OR = 2.04 (0.48–8.66) *p* = 0.33 NS
Non-OCR	69 (88.5)	85 (87.6)	43 (86)	61 (85.9)		48 (92.3)
OCR	9 (11.5)	12 (12.4)	7 (14)	10 (14.1)		4 (7.7)
OR (95%CI) *p*-Value	ref	OR = 1.08 (0.43–2.72) *p* = 0.87 NS	ref	OR = 1.01 (0.35–2.85) *p* = 0.99 NS		OR = 0.51 (0.14–1.87) *p* = 0.31 NS
Parameter	Arterial hypertension (HA)		Diabetes mellitus (DM)			
	No	Yes	Insulin-dependent DM	Insulin-independent DM		Non-diabetics
non-PONV	48 (94.1)	110 (89.4)	45 (84.9)	20 (83.3)		94 (96.9)
overall PONV	3 (5.9)	13 (10.6)	8 (15.1)	4 (16.7)		3 (3.1)
OR (95%CI) *p*-Value	ref	OR = 1.89 (0.52–6.94) *p* = 0.34 NS	OR = 5.57 (1.41–22) *p* = 0.01	OR = 6.27 (1.3–30.2) *p* = 0.02		ref
Non-OCR	46 (90.2)	107 (87)	48 (90.6)	21 (87.5)		84 (86.6)
OCR	5 (9.8)	16 (13)	5 (9.4)	3 (12.5)		13 (13.4)
OR (95%CI) *p*-Value	ref	OR = 1.38 (0.48–3.98) *p* = 0.56 NS	OR = 0.67 (0.23–2) *p* = 0.48 NS	OR = 0.92 (0.24–3.54) *p* = 0.91 NS		ref
Parameter	Motion sickness (MS)		Smoking			
	No	Yes	No		Yes	
non-PONV	147 (91.3)	12 (85.7)	137 (90.1)		22 (95.7)	
overall PONV	14 (8.7)	2 (14.3)	15 (9.9)		1 (4.3)	
OR (95%CI) *p*-Value	ref	OR = 1.75 (0.36–8.62) *p* = 0.49 NS	ref		OR = 0.42 (0.05–3.3) *p* = 0.41 NS	
Non-OCR	140 (87)	14 (100)	22 (95.7)		132 (86.8)	
OCR	21 (13)	0 (0)	1 (4.3)		20 (13.2)	
OR (95%CI) *p*-Value	ref	-	ref		OR = 0.3 (0.04–2.35) *p* = 0.25 NS	
Parameter	Fluid challenge (mL)		Postoperative pain perception			
			NRS ≤ 3		NRS > 3	
non-PONV	996.8 + 312.2 1000 (360)		128 (90.1)		31 (93.9)	
overall PONV	940 + 211.5 1000 (250)		14 (9.9)		2 (6.1)	
OR (95%CI) *p*-Value	OR = 0.99 (0.998–1.001) *p* = 0.49 NS		ref		OR = 0.59 (0.13–2.73) *p* = 0.49 NS	
non-OCR	978.8 + 287.8 1000 (350)		126 (88.7)		28 (84.8)	
OCR	1083.3 + 400.1 1000 (450)		16 (11.3)		5 (15.2)	
OR (95%CI) *p*-Value	OR = 1.001 (0.999–1.003) *p* = 0.14 NS		ref		OR = 1.41 (0.48–4.16) *p* = 0.53 NS	

Results presented as numbers (percentages) for nominal variables, odds ratios OR with 95% CI and *p*-values by Fisher’s exact test. There was a significantly higher risk of PONV for DM groups (*p* < 0.05) compared with non-DM group. Abbreviations: PONV—postoperative nausea and vomiting; OCR—oculocardiac reflex; OR—odds ratio; 95% CI—95% confidence interval of odds ratio; ref—reference group; NS—statistically insignificant differences (*p* > 0.05); mL—milliliters; NRS—numeric pain rating scale.

## Data Availability

Data is contained within the article.

## Supplementary Materials

The following are available online at https://www.mdpi.com/article/10.3390/ph15020237/s1. Table S1: Characteristics of the patients who participated in the study.
