# Supplementary material for: Adverse Events during Vitreoretinal Surgery under Adequacy of Anesthesia Guidance—Risk Factor Analysis
Source: Pharmaceuticals (Basel). 2022 Feb 16;15(2):237. doi: 10.3390/ph15020237 (PMC8879673; doi:10.3390/ph15020237)
Supplement: Supplementary file 1 [file pharmaceuticals-15-00237-s001.zip › pharmaceuticals-1503867-supplementary.pdf]

Table S1. Characteristics of the patients participating in the study

Anthropometric Data		Total *n* = 175 (100%)	GA Group *n* = 35 (20%)	M Group *n* = 35 (20%)	P Group *n* = 35 (20%)	PBB Group *n* = 35 (20%)	T Group *n* = 35 (20%)	*p*-Value
**The results of the one-way analysis of variance (ANOVA)/the Kruskal–Wallis test by ranks**								
Age<br>X ± S<br>M (Rk)	[years]	64.5 ± 11.7<br>66 (13)	65.1 ± 10.8<br>67 (9)	61.9 ± 11.9<br>63 (14)	66.1 ± 9.9<br>67 (8)	66.8 ± 12.1<br>69 (13)	62.7 ± 13.3<br>65 (14)	$p = 0.25$ [a]<br>NS
High<br>X ± S<br>M (Rk)	[cm]	165.8 ± 8.7<br>165 (12)	166.9 ± 8.6<br>168 (14)	168 ± 7.4<br>170 (14)	163.4 ± 8.7<br>160 (12)	165.9 ± 8.3<br>164 (12)	164.7 ± 10.3<br>164 (18)	$p = 0.18$ [a]<br>NS
Weight<br>X ± S<br>M (Rk)	[kg]	77.6 ± 15.9<br>75.5 (17)	83.4 ± 19.8<br>82 (20)	74.7 ± 14.9<br>74 (19)	74.1 ± 13.3<br>74 (22)	78.8 ± 16<br>75 (11)	77.1 ± 13.7<br>80 (21)	$p = 0.19$ [a]<br>NS
BMI<br>X ± S<br>M (Rk)	[kg/m^2]	28.3 ± 5.4<br>27.5 (6.4)	29.9 ± 6.6<br>28.4 (5.3)	26.4 ± 4.6<br>25.3 (5.4)	27.9 ± 5.3<br>27.6 (7.7)	28.6 ± 5.1<br>27.1 (4.4)	28.5 ± 4.9<br>28.4 (7.3)	$p = 0.05$ [a]
**The results of the χ^2 test of independence**								
Gender<br>*n* (%)	Female	97 (55.4)	18 (51.4)	15 (42.9)	24 (68.6)	21 (60)	19 (54.3)	$p = 0.26$ [b]<br>NS
	Male	78 (44.6)	17 (48.6)	20 (57.1)	11 (31.4)	14 (40)	16 (45.7)	
Diabetes Mellitus	Insulin-dependent	53 (30.3)	11 (31.4)	6 (17.1)	12 (34.3)	12 (34.3)	12 (34.3)	$p = 0.45$ [b]<br>NS
	Insulin-independent	45 (25.7)	10 (28.6)	3 (8.6)	12 (34.3)	9 (25.7)	11 (31.4)	$p = 0.11$ [b]<br>NS
**The results of the multiple proportions test**								
BMI<br>*n* (%)	Norm	50 (28.7)	5 (14.3)	15 (42.9)	14 (40)	7 (20.6)	9 (25.7)	$p < 0.05$ [c]
	Overweight	72 (41.4)	18 (51.4)	13 (37.1)	9 (25.7)	19 (55.9)	13 (37.1)	$p = 0.09$ [c]<br>NS
	Obesity	52 (29.9)	12 (34.3)	7 (20)	12 (34.3)	8 (23.5)	13 (37.1)	$p = 0.41$ [c]<br>NS

Results are presented as means ± standard deviations and medians (interquartile ranges). a One-way analysis of variance (ANOVA)/the Kruskal–Wallis test by ranks. Nominal data are presented as numbers (percentages). b $\chi 2$ test of independence. c Multiple proportions test. BMI, body mass index. MAP—Mean Arterial Pressure; SAP—Systolic Arterial Pressure; DAP—Diastolic Arterial Pressure; PBB—preprocedural peribulbar block group; M group—metamizole group; P—Paracetam
